# A Manual of Practical Therapeutics

**Published:** 1886-10

**Authors:** 


### Reviews.

**A Manual of Practical Therapeutics**, considered with reference to articles of the Materia Medica. By EDWARD JOHN WARING, C. I. E., M.D., Fellow of the Royal College of Physicians, London. Edited by DUDLEY W. BUXTON, M. D., B. S., London, member of the Royal College of Physicians; assistant to the Professor of Medicine at the University College, London, etc. Fourth edition. Philadelphia: P. Blackiston, Son & Co., No. 1012 Walnut street. 1886.

"During the two and thirty years which have elapsed since the first edition of this manual made its appearance, therapeutics, both in theory and practice, have undergone many important changes and advanced with rapid strides in common with other branches of medical science." This work has been revised from time to time during this period, and is now brought down to date. Many subjects of less interest being omitted or curtailed considerably, so that the size of the present edition is about the same as former numbers of the work. The work of review was carried on by the editor himself until infirmities of advancing years obliged him to desist. He continued it as far as the article on quinine. After this the work was performed by Dr. Burton, who is well qualified to continue it. "He has also written the articles on mineral waters, malt, pepsin, peptonized foods, oleic acid, salicylic acid, and the salicylates, nitro-glycerine, nitrites, sulphites, etc. These contributions cannot fail to give increased value and interest to this edition." The work is a safe guide, and a valuable addition to the physician's library.

